# Factors That Affect the Likelihood of Undergoing Cosmetic Procedures Among the General Population of Western Region, Saudi Arabia: A Cross-Sectional Study

**DOI:** 10.7759/cureus.49792

**Published:** 2023-12-01

**Authors:** Eyad E Sindi, Raghad Y Shosho, Maria A AlSulami, Abrar A Alghamdi, Manal D Alnemari, Waref H Felemban, Fahad Aljindan, Mohammed Alharbi, Lamees Altamimi, Rehab S Almajnuni

**Affiliations:** 1 Department of Medicine and Surgery, College of Medicine, Umm Al-Qura University, Makkah, SAU; 2 Plastic and Reconstructive Surgery, King Abdullah Medical City, Makkah, SAU; 3 Aesthetic Plastic Hand, Burn, and Reconstructive Surgery, ‏King Abdullah Medical City, Makkah, SAU; 4 Plastic Surgery, King Saud University, Riyadh, SAU; 5 Family Medicine, Al-shara’e2 Primary Health Care, Makkah Healthcare Cluster, Makkah, SAU

**Keywords:** kingdom of saudi arabia (ksa), aesthetic facial surgery, general public, plastic surgery, cosmetic surgery

## Abstract

Background and aim: Cosmetic procedures are surgical and non-surgical procedures that improve and reshape body or facial structures to improve someone’s appearance, self-esteem, and confidence. In recent years, these procedures have gained more popularity, and both the number of procedures performed and the cosmetic procedure market are growing dramatically worldwide. The objective of our research is to carry out a cross-sectional investigation to assess the factors that affect the likelihood of undergoing cosmetic surgery in the Western Region of Saudi Arabia.

Methods: In this study, a descriptive, cross-sectional methodology was employed. The intended sample includes residents of Makkah, Medina, Jeddah, and Al-taif cities who are 18 years of age or older, representing the general population. Data collection was carried out through an online questionnaire created using Google Forms, which was disseminated electronically via social media platforms. The questionnaire gathered demographic information and questions that evaluate the time spent on social media, likelihood of having cosmetic surgery, cosmetic surgery experience, social media exposure, personal experience, and self-rating of attractiveness.

Results: The study included a total of 507 participants, with 389 (76.7%) being female and 118 (23.3%) being male. In our sample, there was a significant correlation between the likelihood of undergoing cosmetic surgery in women who have a longer exposure to media and lower self-rating of attractiveness. For men, media exposure and previous cosmetic procedures were significantly affecting their likelihood of undergoing cosmetic procedures.

Conclusions: Females who had a lower self-rating of attractiveness and a longer exposure to social media were more likely to undergo a cosmetic procedure. However, to gain a more comprehensive understanding, further research should be conducted.

## Introduction

Cosmetic procedures encompass surgical and non-surgical techniques designed to enhance and refine body or facial features, ultimately contributing to an individual’s appearance, self-esteem, and self-confidence [1]. These procedures range from prominent interventions, such as breast augmentation, liposuction, and rhinoplasty, to more minimally invasive options, including Botox and filler injections [2].

In recent years, there has been a notable surge in the popularity of these procedures, resulting in a remarkable global increase in the volume of procedures conducted [3,4]. In light of this transformation, practitioners are seeking to excel within their by gaining a deeper understanding of public preferences concerning cosmetic procedures.

Prior research examining these variables encompasses a study undertaken in the United Kingdom. This study unveiled that among non-invasive procedures, the most coveted encompassed teeth whitening, body hair removal, facial hair removal, skin texture and appearance enhancement, and reduction of acne scarring. Among invasive procedures, the prevailing preferences included weight loss, anti-wrinkle treatments, mammoplasty, rhinoplasty, eye bag removal, and height increase. Notably, within the same study, a higher likelihood of pursuing cosmetic procedures was observed especially among women participants who reported a lower self-rating concerning their attractiveness [5].

Comprehending the impact of social media on individuals’ choices regarding cosmetic surgery is paramount. A study from the past highlights that a significant proportion (65.7%) of patients attending plastic surgery clinics in Saudi Arabia drew inspiration from images of aesthetic procedures shared on the social media profiles of aesthetic surgeons [6]. Research by McLean et al. revealed that teenage girls who frequently posted selfies on social media exhibited an elevated body dissatisfaction. Furthermore, frequent consumption of selfies correlated with diminished self-esteem and reduced life satisfaction [7]. These findings underscore the influence of social media on people’s decisions to explore cosmetic surgery options.

According to researchers, no studies have been conducted in Saudi Arabia to evaluate the propensity for undergoing cosmetic surgery and the specific procedures most coveted. Consequently, they posit that such a survey holds immense significance for physicians, as it would provide them with a deeper understanding of the driving forces behind patients seeking their assistance and shed light on the procedures more likely to be in demand.

## Materials and methods

Participants

We recorded 507 participants, with the majority (76.7%) being females. The participants’ ages ranged from 18 to over 50. Of the participants, 71.8% held at least a bachelor’s degree. The majority 476 (93%), were of Saudi nationality. Regarding marital status, 348 individuals (68.6%) were single, while the remaining participants were married or divorced. A significant portion of the participants (62.9%) reported a monthly income of less than 5,000 SR.

Procedure

The present study is a cross-sectional examination of the adult general population of the Western Region of Saudi Arabia. OpenEpi version 3.0 (Open Source Epidemiologic Statistics for Public Health, www.OpenEpi.com) was utilized to determine the minimum required sample size for this inquiry, factoring in the following aspects: The estimated population stands at approximately 8,325,304 individuals (according to the General Authority of Statistics) [8], with a 95% confidence interval (CI) and an expected frequency of 50%. The computed sample size was 385 participants, subsequently increasing to 400 to account for potential data loss. The participants were enlisted through convenience sampling. The questionnaire was designed using Google Forms (Google, USA), and the items were chosen and adjusted based on a review of existing literature [5]. It was disseminated in Arabic online, reaching the target population through social media applications over two weeks. The participants were duly informed about the study’s objectives and voluntarily took part. Prior to completing the questionnaire, informed consent was acquired.

Measures

Sociodemographic Information

The participants were requested to provide details regarding their age, gender, marital status, level of education, nationality, employment status, and monthly income.

Self-Assessment of Attractiveness

The participants were prompted to assess their attractiveness using a seven-point scale (with 7 indicating “very attractive” and 1 indicating “unattractive”) [5].

Personal Experience with Cosmetic Surgery

The participants were asked about any previous plastic surgery experiences. If they had, they were also asked to specify the procedure(s) they underwent. In addition, the extent of their exposure to cosmetic surgery was gauged by the number of individuals they knew who had undergone such procedures. This was rated on a five-point scale (1 = none, 2 = 1-2 persons, 3 = 3-9 persons, 4 = 10 or more persons, 5 = unsure) [5].

Impact of Social Media Exposure on Cosmetic Procedure Desire

The participants were initially prompted to estimate their daily social media usage time from five options (1 = <1 hour, 2 = 1-2 hours, 3 = 2-3 hours, 4 = 3-4 hours, 5 = >4 hours). Subsequently, they were asked to rate the frequency with which social media influenced their desire to undergo a cosmetic procedure. Ratings were provided on a four-point scale (1 = never, 2 = rarely, 3 = often, 4 = always).

Scale for Likelihood of Undergoing Cosmetic Surgery [5]

This scale was modified under the guidance of a practicing consultant plastic surgeon in Saudi Arabia. The participants were presented with a list of 25 prevalent cosmetic surgery procedures and were then asked to assess the likelihood of undergoing each procedure if given the opportunity. Ratings were done on an eight-point scale ranging from 0 to 7, where 0 indicated no inclination for change under any circumstances and 7 indicated a strong willingness to undergo the procedure.

Data analysis

The data were collected, reviewed, and subsequently inputted into IBM SPSS Statistics for Windows, version 27 (released 2012; IBM Corp., Armonk, New York, United States). All statistical methodologies employed were two-tailed, with an alpha level of 0.05 set for significance, considering a P-value of less than or equal to 0.05. Descriptive analysis involving frequency and percentage was applied to categorical variables, while the mean and standard deviation were employed for participants’ cosmetic procedure desire scores (ranging from 0 to 7). The “general likelihood” of undergoing cosmetic surgery was used to evaluate predictors for the desire to undergo a cosmetic procedure through multilevel stepwise regression analyses. The predictor variables employed in this analysis encompassed sex, age, media exposure, self-assessment of attractiveness, and prior experiences with cosmetic surgery.

Ethical approval

The study was approved by the Institutional Research Board at Umm Al-Qura University, Makkah City, Saudi Arabia (HAPO-02-K-012-2023-03-1535). All study participants were informed of the study’s objectives and were assured that their responses would be kept confidential. 

## Results

A total of 507 eligible participants completed the study questionnaire. The participants’ ages ranged from 18 to over 50 years, with a mean age of 26.5 ± 13.9 years old. Specifically, 389 (76.7%) of the participants were females, 348 (68.6%) were single, and 138 (27.2%) were married. In terms of educational level, 117 (23.1%) had a secondary level of education or below, 364 (71.8%) had a university level of education, and 26 (5.1%) held a post-graduate degree. Among the participants, 476 (93.9%) were of Saudi nationality. Concerning their occupations, 267 (52.7%) were students, and 144 (28.4%) were employees. A monthly income of less than 5,000 SR was reported by 319 (62.9%), while 89 (17.6%) had a monthly income ranging from 5,000 to 10,000 SR (Table 1).

**Table 1:** Personal characteristics of the study participants in Western Region, Saudi Arabia.

Personal characteristics	No	%
Age in years		
18-29	349	68.8%
30-39	56	11.0%
40-49	64	12.6%
50+	38	7.5%
Gender		
Male	118	23.3%
Female	389	76.7%
Marital status		
Single	348	68.6%
Married	138	27.2%
Divorced/widow	21	4.1%
Educational level		
Secondary/below	117	23.1%
University/diploma	364	71.8%
Post-graduate	26	5.1%
Nationality		
Saudi	476	93.9%
Non-Saudi	31	6.1%
Work		
Not working/retired	85	16.8%
Student	267	52.7%
Employee	144	28.4%
Free works	11	2.2%
Monthly income		
<5000 SR	319	62.9%
5000-10000 SR	89	17.6%
11000-20000 SR	79	15.6%
21000-30000 SR	10	2.0%
>30000 SR	10	2.0%

**Figure 1:**
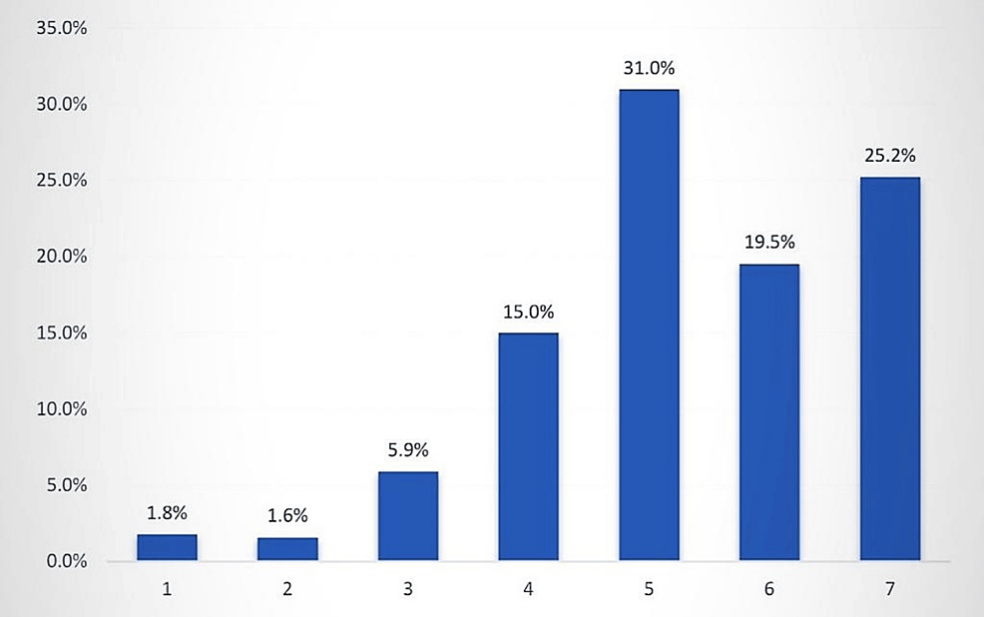
Self-perceived attractiveness among the study participants in Western Region, Saudi Arabia.

Approximately 31% of the participants rated themselves at 5 out of 7 in terms of attractiveness, while 19.5% rated themselves at 6 out of 7, and 25.2% rated themselves at 7 out of 7. The mean rating was 5.3 ± 1.4 out of 7, constituting 75.7% of the responses (Figure 1).

A total of 55 participants (10.8%) had previously undergone a cosmetic procedure. Moreover, 140 participants (27.6%) reported knowing one or two individuals who had undergone cosmetic procedures, 154 participants (30.4%) knew three to nine individuals, while 132 participants (26%) had never known anyone who had undergone any cosmetic procedure (Table 2).

**Table 2:** Personal and family history of undergoing cosmetic procedure.

Cosmetic procedures	No	%
Have you ever undergone any cosmetic procedure?		
Yes	55	10.8%
No	452	89.2%
The number of people you know personally who have had any cosmetic procedures		
No one	132	26.0%
1-2 persons	140	27.6%
3-9 persons	154	30.4%
10/more	49	9.7%
Not sure	32	6.3%

Among the respondents, 205 (40.4%) reported using social media platforms for more than four hours daily, 98 (19.3%) used them for three to four hours daily, and 113 (22.3%) used them for two to three hours daily. In terms of the influence of social media on the desire for cosmetic procedures, 145 participants (28.6%) indicated rare influence, 114 participants (22.5%) mentioned frequent influence, 31 participants (6.1%) reported constant influence, while 217 participants (42.8%) stated that social media never influenced them (Table 3).

**Table 3:** Social media use and its influence on desire to undergo a cosmetic procedure.

Social media	No	%
Duration you spend on social media daily		
<1 hour	12	2.4%
1-2 hours	79	15.6%
2-3 hours	113	22.3%
3-4 hours	98	19.3%
>4 hours	205	40.4%
Does social media influence your desire to undergo a cosmetic procedure?		
Never	217	42.8%
Rarely	145	28.6%
Often	114	22.5%
Always	31	6.1%

For the face and head, the highest mean score was observed for anti-wrinkle (forehead) injection (2.1 out of 7), followed by rhinoplasty (1.9 out of 7). Among the female participants, the highest mean scores were recorded for anti-wrinkle (forehead) injection (2.4) and lip augmentation (2.1), while for the male participants, the highest mean score was for rhinoplasty (1.4). Regarding body procedures, the highest score was attributed to liposuction or fat removal (2.1), followed by abdominoplasty (1.9). Among women, the highest scores were for liposuction or fat removal (2.3) and abdominoplasty (2.1); for men, the scores were 1.6 and 1.4, respectively. Breast uplift and genital cosmetic surgery received mean scores of 1.5 out of 7 among women, whereas breast reduction scored 1.3 out of 7 (Table 4).

**Table 4:** Cosmetic procedure items and mean scores with standard deviations by gender. SD: standard deviation

Body part/procedure*	Total		Gender			
			Male		Female	
	Mean	SD	Mean	SD	Mean	SD
Face and head						
Anti-wrinkle (forehead) injection	2.1	2.2	1.1	1.7	2.4	2.3
Injection for nose reshaping (rhinoplasty)	1.9	2.2	1.4	1.9	2.0	2.2
Injection for brow or forehead lift	1.5	1.9	0.9	1.4	1.7	2.0
Lip injection for augmentation	1.7	2.1	0.7	1.1	2.1	2.3
Cheek injections to increase or tighten their size	1.3	1.8	0.7	1.3	1.5	1.9
Injection for mentoplasty	1.5	1.9	0.9	1.5	1.6	2.0
Injection for ear pinning (otoplasty)	0.9	1.3	0.9	1.5	.9	1.3
Face and neck lift (surgically)	1.2	1.8	0.9	1.6	1.3	1.8
Removing excess skin from the upper or lower eyelid	1.3	1.8	0.9	1.5	1.4	1.9
Body (women and men)						
Liposuction or fat removal	2.1	2.4	1.6	2.1	2.3	2.4
Reshaping of body with fatty deposits	1.3	1.8	0.8	1.4	1.5	1.9
Nipple reshaping	0.9	1.4	0.7	1.2	1.0	1.4
Umbilicoplasty	0.8	1.3	0.7	1.2	0.9	1.3
Waist sculpting	1.5	2.0	0.8	1.4	1.7	2.2
Buttock lift or implants	1.4	1.9	0.8	1.4	1.5	2.0
Thigh lift (thighplasty)	1.5	2.0	1.2	1.8	1.6	2.0
Tummy tuck (abdominoplasty)	1.9	2.3	1.4	2.1	2.1	2.4
Tummy tuck with back (belt lift)	1.5	2.1	1.2	1.8	1.6	2.1
Length of arms	1.6	2.2	1.1	1.8	1.8	2.2
Body (women only)						
Breast enlargement	1.3	1.8			1.3	1.8
Breast reduction	1.0	1.5			1.0	1.5
Breast uplift	1.5	2.0			1.5	2.0
Genital reshaping	1.5	2.0			1.5	2.0
Body (men only)						
Breast reduction	1.0	1.4	1.0	1.4		
Reshaping to highlight abdominal muscles	1.0	1.6	1.0	1.6		

In Model 1, which encompassed sex, age, media exposure, self-ratings of attractiveness, and vicarious experience with cosmetic surgery, a significant prediction was observed for the likelihood of undergoing cosmetic surgery (P < 0.05, R^2^ = 0.13). Upon examining individual weightings within this step, three variables emerged as significant predictors of this “general likelihood”: sex (B = 0.41, P < 0.05), with women showing a higher tendency than men; self-ratings of attractiveness (B = -0.20, P < 0.05), with lower ratings correlating to a higher likelihood of undergoing surgery; and media exposure (B = 0.08, P < 0.05). In the model exclusively for women, media exposure (B = 0.014) and self-attractiveness ratings (B = -0.22) were significant predictors. For the men-only model, media exposure (B = 0.012) and a history of undergoing cosmetic procedures (B = 0.016) significantly influenced their likelihood of considering cosmetic procedures (Table 5).

**Table 5:** Results of the multilevel regression analyses.

	β	β	R	R^2^	Adj R^2^	Delta F
General likelihood (Step 1)						
Sex	0.75	0.41 *	0.36	0.13	0.19	10.2 *
Age	0.01	-0.07				
Media exposure	0.06	0.08 *				
Ratings of self-attractiveness	-0.14	-0.20 *				
Undergone cosmetic procedure	0.01	0.06				
General likelihood (women only)						
Age	0.03	0.08	0.47	0.22	0.33	6.4 *
Media exposure	0.10	0.14 *				
Ratings of self-attractiveness	-0.17	-0.22 *				
Undergone cosmetic procedure	0.09	0.13				
General likelihood (men only)						
Age	0.04	0.07	0.24	0.06	0.14	4.3 *
Media exposure	0.08	0.12*				
Ratings of self-attractiveness	-0.09	-0.13				
Undergone cosmetic procedure	0.11	0.16 *				

## Discussion

The popularity of aesthetic procedures has gained a substantial increase. In a local context, among the 25 countries with the highest rate of cosmetic operations, Saudi Arabia was ranked 22nd [9,10]. Various cultural, societal, and individual variables, along with the influence of social media, collectively impact individuals’ choices to undergo cosmetic procedures. This study aims to pinpoint the pivotal factors that shape people’s decisions to pursue cosmetic procedures in the Western Region of Saudi Arabia.

Our findings reveal that only a minority (10.8%) of the respondents had undergone cosmetic procedures, a figure lower than that reported in a previous study conducted among female students in Riyadh, Saudi Arabia [11]. An expected result emerged, showing that women were more inclined to undergo cosmetic surgery than their male counterparts, with a statistically significant P-value (B = 0.41, P < 0.05). These results are in line with prior research [12].

Another notable finding is that participants with lower self-esteem ratings are more inclined to contemplate undergoing aesthetic procedures. This discovery aligns with the results of a previously published study that identified an aspiration for enhanced self-confidence as a significant motivator for opting for cosmetic procedures among Saudi females [10]. Furthermore, this trend is corroborated by data from Iran, where a study involving 80 females and 20 males indicated that self-improvement was the primary driving factor behind the inclination to undergo cosmetic surgery [13]. This phenomenon could be attributed to social media influencers’ influence and the advertisements proliferating on social media platforms. However, the amplified presence on social media may harm one’s self-perception.

When the participants in the current study were queried about their preferred types of procedures, the highest mean score among females for facial and head treatments was attributed to anti-wrinkle (forehead) injection (2.4), closely followed by lip augmentation (2.1). This observation finds support in the International Survey on Aesthetic/Cosmetic Procedures, which identified Botox injections as the most prevalent non-surgical procedure conducted in aesthetic clinics worldwide (38.4%), followed by fillers (23.8%) [14]. A separate study in the United States also highlighted Botox injections and dermal fillers as the most frequently performed non-surgical procedures [15]. This preference could stem from patients’ inclination toward less invasive and more cost-effective alternatives to surgery, thus explaining the surge in popularity of these procedures.

Regarding body procedures, insights from the International Survey on Aesthetic/Cosmetic Procedures indicated that women in the United States and other countries tend to opt for invasive procedures like liposuction [14]. Our study echoes this trend, showing that the highest score was awarded to liposuction (2.3), followed by abdominoplasty (2.1) for both females and males. Similar trends were highlighted by the American Society for Aesthetic Plastic Surgery, which reported liposuction as the most sought-after surgical procedure in 2020, trailed by breast augmentation [15]. The increasing popularity of liposuction can likely be attributed to its effectiveness, well-tolerated nature, and positive impact on post-operative quality of life [16].

The present study reveals a significant relationship between social media exposure and the likelihood of undergoing cosmetic procedures. This result contradicts the findings of Brown et al. [5], whose research indicated that media exposure had no impact on the likelihood of such procedures for either gender. In a study conducted among 816 female university students in Riyadh, Saudi Arabia, it was observed that social media played a role in influencing their contemplation of cosmetic procedures. Interestingly, most participants reported spending more than five hours daily on social media (53.2%; P = 0.026) [11]. Our study’s results are consistent with this, with 205 participants (40.4%) indicating that they use social media platforms for over four hours daily.

Holland et al. [17] also found a correlation between extensive social media usage, increased exposure to appearance-related content, and heightened body image concerns. A targeted study by Al-Saiari and Bakarman [18] reported that nearly half of the participants who had undergone cosmetic surgery (46.2%) believed that mass media did influence decisions about such procedures. Similarly, another study involving 118 women aged 18 to 29 highlighted that exposure to images of individuals with cosmetic modifications impacted young women’s desire for cosmetic surgery, particularly those who were heavy social media users, followed numerous accounts and experienced dissatisfaction with their appearance [19].

Consistent with these findings, it was noted that 65.7% of patients in Saudi Arabia were motivated to visit plastic surgery clinics due to before-and-after photos of plastic surgery posted on aesthetic surgeons’ social media pages [6]. This trend could be attributed to women’s aspiration for their appearance to align with the female beauty ideals propagated by the media.

Strengths and limitations

The current study represents the inaugural attempt to assess the likelihood of undergoing cosmetic procedures within the population of the Western region of Saudi Arabia. Nevertheless, it is important to acknowledge that the findings from this specific context might not readily apply to the wider Saudi population.

## Conclusions

The primary objective of this study was to evaluate the probability of opting for cosmetic surgery and to identify the most preferred procedure among participants. Our findings underscore that individuals who are female, hold a lower self-assessment of their attractiveness, possess a history of prior cosmetic procedures, and engage with social media for an extended period are more inclined to consider cosmetic interventions. Among women, the most coveted procedures for the head and face included forehead anti-wrinkle injections and lip augmentation, while rhinoplasty topped the list for men. Regarding body procedures, both men and women expressed a desire for liposuction and abdominoplasty. Encouragement is extended to future research endeavors that utilize validated questionnaires to assess the likelihood of pursuing cosmetic procedures within other demographic populations.
